# Differential modulation of human beta-defensin-3 expression in human oral epithelial cells by HPV oncoproteins E6 and E7: potential implication in oral cancer

**DOI:** 10.1186/1750-9378-7-S1-O11

**Published:** 2012-04-19

**Authors:** Ge Jin, Emeka Innocent, Brian Chow, Jing Bian, Jacob Dayan, Thomas McCormick, Aaron Weinberg

**Affiliations:** 1 Department of Biological Sciences, Case Western Reserve University School of Dental Medicine, Cleveland, OH, USA; 2 School of Medicine, Case Western Reserve University, Cleveland, OH, USA; 3 Department of Dermatology, Case Western Reserve University School of Medicine, University Hospitals Case Medical Center, Cleveland, OH, USA

## Background

Human papillomaviruses (HPVs) are small, non-enveloped DNA viruses that infect stratified squamous mucosal and cutaneous epithelia, causing diseases ranging from benign warts to invasive tumors. Failure of the immune system to detect and clear persistent HPV infections frequently leads to the development of oral warts and cancer. HPV infection has been etiologically linked with oral warts and a subset of oral squamous cell carcinoma, particularly in HIV infected patients. The incidence of HPV-related oral lesions is increased in HIV+ subjects on highly active antiretroviral therapy (HAART). We previously showed that tumor cells in oral carcinoma in situ (CIS) lesions overexpress human beta-defensin-3 (hBD-3), an antimicrobial peptide with immunomodulatory capabilities. Expression of hBD-3 in CIS contributes to the local pro-tumor immune response by selectively chemoattracting tumor-associated macrophages and by enhancing tumor development and progression.

## Results

To elucidate mechanisms by which high-risk HPV could evade immune detection and clearing via infected epithelial cells, we investigated if oncoproteins E6 and E7 derived from high-risk HPV-16 modulate the innate immune response of infected epithelial cells and the role of HPV-induced gene expression in orchestrating local immunity. We have found that cancer cells of HPV-related oral and oropharyngeal squamous cell carcinoma biopsies overproduce hBD-3. Introduction of an expression vector producing HPV-16 E6 or E7 oncogene into oral squamous cancer cell lines or primary oral epithelial cells increases the levels of hBD-3 mRNA and peptide. However, E6 derived from the low-risk HPV-11 is significantly less potent in promoting hBD-3 expression. Combination of oncogenic E6 and E7 in oral epithelial cells also shows reduced induction of hBD-3. Furthermore, the transactivity of an hBD-3 luciferase promoter construct is differentially stimulated by oncogenic E6 and E7 compared with MEKK1, a known inducer of hBD-3 expression. Although the pharmacological inhibitors for MAPK and PI3K reduce the transactivity of a 2.5 kb hBD-3 promoter reporter, they do not exhibit the inhibitory effect on the promoter reporter containing a 450 bp 3’-regulatory region. These data suggest that high-risk and low-risk early genes of HPV differentially modulate hBD-3 expression in oral epithelial cells.

## Conclusion

Our results suggest that oncoproteins of high-risk HPV strains induce higher levels of hBD-3 expression compared with early genes of low-risk HPV. The oncogenic E6 and E7 genes may contribute to overexpression of hBD-3 in the early oral lesion, which then leads to recruitment of tumor-associated macrophages to further develop and promote the progression of cancer.

